# Which Proteins? The Challenge of Identifying the Protective Antigens for Next-Generation Capripoxvirus Vaccines

**DOI:** 10.3390/vaccines13030219

**Published:** 2025-02-22

**Authors:** Mahder Teffera, Hani Boshra, Timothy R. Bowden, Shawn Babiuk

**Affiliations:** 1 Canadian Food Inspection Agency, National Centre for Foreign Animal Disease, Winnipeg, MB R3E 3M4, Canada; mteffera@student.ubc.ca; 2 Department of Pathology, Fundamental and Applied Research for Animals and Health (FARAH), Faculty of Veterinary Medicine, University of Liège, 4000 Liège, Belgium; hboshra@yahoo.com; 3 Commonwealth Scientific and Industrial Research Organisation (CSIRO), Australian Centre for Disease Preparedness (ACDP), East Geelong, VIC 3219, Australia; timothy.bowden@csiro.au; 4 Department of Immunology, Max Rady College of Medicine, University of Manitoba, Winnipeg, MB R3E 0T5, Canada

**Keywords:** vaccine, capripoxvirus, lumpy skin disease, sheeppox, goatpox

## Abstract

Sheeppox, goatpox, and lumpy skin disease continue to negatively impact the sheep, goat, and cattle industries in countries where these diseases are present and threaten to spread into new regions. Effective vaccines are available for disease control and eradication. However, commercial vaccines are based on live attenuated virus isolates and therefore it is not currently possible to differentiate between infected and vaccinated animals (DIVA), which severely limits the use of these vaccines in countries that are free from disease and at risk of an incursion. The development of next-generation vaccines, including recombinant protein, viral-vectored, and mRNA, has been limited due to the lack of understanding of the protective antigen(s) of capripoxviruses. The complexity of capripoxviruses, with up to 156 open reading frames, makes the identification of protective antigen(s) difficult. This paper identifies the most promising antigens by first considering the membrane-associated proteins and then further selecting proteins based on immunogenicity and their role in immunity by comparing them to known orthopoxvirus homologues. From the 156 potential antigens, 13 have been identified as being the most likely to be protective. Further evaluation of these proteins, as immunogens, would be required to identify the optimal combination of immunodominant antigen(s) for the development of next-generation capripoxvirus vaccines.

## 1. Introduction

Lumpy skin disease virus (LSDV), together with sheeppox virus (SPPV) and goatpox virus (GTPV), is a member of the genus *Capripoxvirus* in the family *Poxviridae* [1]. Capripoxviruses are large double-stranded DNA viruses that have a high genetic similarity (96–99% identity at the nucleotide level) between members of the genus [2,3]. The number of open reading frames is 156 for LSDV [2] and 147 for SPPV and GTPV [3]. There are no recognized serotypes; therefore, the viruses cannot be distinguished serologically [4]. Capripoxviruses can only be differentiated using molecular tests, including sequencing. LSDV primarily infects cattle, whereas SPPV and GTPV infect sheep and goats, respectively; most isolates display a host preference [5].

SPPV and GTPV cause severe disease characterized by fever and skin lesions, with high morbidity (100%) and mortality (up to 100%) rates [6]. Lumpy skin disease also causes fever and skin lesions; although morbidity can vary (50–100%), the mortality rate is generally low (0–10%) compared to sheeppox and goatpox [7]. LSDV is primarily transmitted through mechanical transmission by vectors, which makes control of the disease difficult, with eradication only being successful when effective vaccination campaigns were part of the overall control response [8,9]. In contrast, SPPV and GTPV can be eradicated through stamping out and restricting the movement of animals [10,11]. The vaccines widely used to control capripoxviruses are based on live attenuated virus strains [7].

Historically, LSDV has been geographically limited to Africa; however, that is no longer the case, as lumpy skin disease has dramatically increased its geographic range into the Middle East and some parts of Eastern Europe where it was eradicated through mass vaccination [12,13]. Furthermore, it has spread to most countries in Asia and currently poses a threat of entering Australia from Indonesia [14]. SPPV and GTPV are endemic in Central and North Africa, the Middle East, India, and Central Asia, with infrequent epidemics in some European countries such as Russia, Bulgaria, and Greece [15,16,17]. There have also been recent outbreaks of SPPV in Cyprus and Spain [18]. Capripoxviruses have a significant socioeconomic impact in endemic countries and further spread poses a threat that requires active intervention [19,20,21].

## 2. Currently Used Vaccines

Live attenuated vaccines for control of lumpy skin disease can be either homologous, if derived from LSDV, or heterologous if derived from SPPV or GTPV [22]. Although both homologous and heterologous vaccines have been used in the field, only homologous vaccines have resulted in the successful eradication of LSDV, as illustrated in Europe [22]. These live attenuated vaccines demonstrate that it is possible to protect cattle [23]. Although effective in cattle, these vaccines can have some side effects, including injection site reactions that may be coupled with lowered milk production; these side effects have been termed the ‘Neethling response’ [23,24]. Furthermore, antibody response elicited from live attenuated vaccines cannot be differentiated from that in animals that have been infected naturally [7].

## 3. Rationale for Identification of Protective Antigens

The mechanism of protection by live attenuated LSDV vaccines is a combination of cell-mediated and humoral immune responses, neither of which is well understood; vaccinated cattle can be protected with live attenuated vaccines in the absence of detectable neutralizing antibodies [23]. The role of antibodies in protecting animals from capripoxvirus disease has been demonstrated using serum transfer experiments, which showed that sheep can be protected from SPPV [25]. While there could be protection in animals after vaccination without detectible neutralizing antibodies, the sufficient protection provided by passive serum transfer experiments shows that, conversely, antibodies alone can also be protective.

More recently, inactivated lumpy skin disease vaccines have been shown to be effective in protecting cattle from LSDV in experimental trials [24,26], and were also able to protect sheep against SPPV [27]. Additionally, a comparison of the duration of protection elicited from live attenuated and inactivated LSDV vaccines in cattle was performed with the inactivated vaccine providing protection for 6 months compared to 18 months for the live attenuated vaccine [28]. For killed vaccines, protection is based mainly on neutralizing antibodies. These killed vaccines primarily contain the intracellular mature virion (IMV) form of the virus and not the extracellular enveloped virion (EEV) form [29]. Moreover, neutralization assays to measure antibody responses are performed mostly with the IMV. These studies together not only demonstrate that the presence of capripoxvirus-neutralizing antibodies can be protective, but that viral antigens on the surface of the IMV as well as EEV are potentially protective antigens. This has also been previously shown in vaccinia virus (VACV) [30], a homologous virus in the *Orthopoxvirus* genus.

Several studies have proposed immunoinformatics-based approaches to develop an LSDV vaccine [31,32,33]. The vaccines proposed in these studies contain both predicted T and B cell epitopes to generate broad immunity. However, these predicted epitopes have not been validated to demonstrate that these epitopes are immunogenic. Additionally, the predicted B cell epitopes have not been demonstrated to be able to neutralize capripoxvirus. The inability of currently used immunoinformatics approaches to identify protective antigens that elicit neutralizing antibodies is a major impediment in successfully using these tools.

Unfortunately, the protective antigen(s) are not currently known for capripoxviruses [21], unlike for many simpler viruses such as SARS-CoV-2, Rift Valley fever, and peste des petits ruminants. Once identified, the protective antigen(s) could be used to develop vaccines in different vaccine platforms (including subunit, mRNA, or viral-vectored). All of these platforms have been shown to be effective in humans, with mRNA and viral-vectored vaccines inducing both cellular and humoral immune responses [34,35]. Therefore, these vaccine platforms have high potential for the development of novel vaccines against capripoxviruses.

## 4. Selection of Potential Protective Antigens

**Figure 1:**
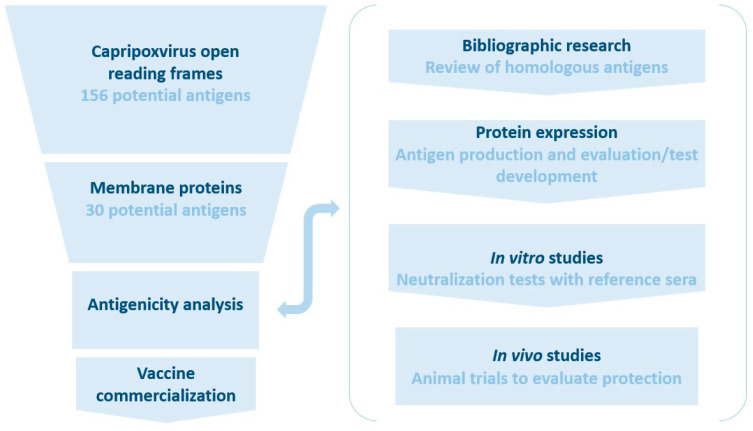
Visual representation of the selection process to find candidate antigens for vaccines. Capripoxviruses have multiple membrane-associated antigens that could be possible protective antigens. These membrane proteins can be further selected for immunogenic antigens and then for antigens that have been shown to provide some measure of immunity against infection.

Since capripoxviruses encode up to 156 proteins, the identification of the protective antigen(s) can be a difficult task. A method or algorithm is required to initially screen the number of antigens into a smaller number of candidates, which would ideally be proven by performing laboratory and animal experiments to show the protective ability of the proposed antigens along with the expression of the candidate antigens to develop serological assays to detect antibody response (Figure 1). The lack of resources in studying animal viruses in general, especially those that have predominantly affected developing countries, is potentially the reason capripoxvirus antigens have not been characterized compared to other poxviruses that are higher-consequence pathogens [36]. Therefore, it is important to narrow down potentially protective antigens with available resources and published information [37].

To identify potentially protective antigens, the first screening step is to identify membrane-associated proteins (Table 1). Poxviruses have two distinct infectious forms: IMV and EEV, which are involved in host viral transmission and long-range dissemination, respectively; therefore, it is important to consider surface antigens present on both the IMV and EEV [38,39]. The next step would be to identify which of these antigens are immunogenic following infection in animals. Virus neutralization can potentially be achieved by prevention of virus replication or inhibition of cell entry through the binding of neutralizing antibodies to surface proteins. However, immunogenicity is not guaranteed by neutralization determinant antigens [40,41]. Using available immunoinformatics tools to analyze the predicted antigenicity of all membrane-associated proteins can also be utilized as another factor to further characterize the proteins. One such tool is VaxiJen, a free online software that has a reported 70–89% accuracy in predicting protective antigens using an alignment-independent algorithm based on protein sequence and amino acid properties [42]. A structural analysis of the potential antigens to determine putative transmembrane regions and the likelihood of exposed epitopes can also be used to further narrow down the list. As the surface antigens studied in capripoxviruses are limited, the next step is to compare these antigens to their homologues from other poxviruses with respect to protection against disease (Table 2). Surface antigens that are not well characterized in other poxvirus homologues are also a potential avenue to pursue after exhausting the initial list of characterized antigens (Table 3).

Several of these surface proteins have been expressed and shown to be detected by specific antibodies in capripoxvirus-infected animals. The identification of these immunogenic proteins allows us to identify potentially protective antigens. Some published examples in capripoxviruses are as follows: LSDV ORF074 [67,68]; LSDV ORF123 and 126 [69]; and SPPV ORF60, 117, and 122 [70]. Due to the variability of proteins on the surface of capripoxviruses, there is a considerable probability that vaccination with more than one antigen is required to result in virus neutralization, especially antigens from both the IMV and EEV forms of the virus [47]. To test this theory, sera generated from vaccines comprising individual antigens can be mixed and evaluated in different combinations to determine the level of virus neutralization elicited from the different pools. This can also be tested through immunization with combinations of target antigens, similar to VACV experiments in mouse models [71]. Moreover, these vaccines should be evaluated for DIVA capability using existing ELISAs or through the development of new ELISAs based on the novelty of the antigens [23].

## 5. Candidate Proteins

### 5.1. LSDV ORF024 (VACV F9L)

F9, a 23.8 kDa protein expressed from the LSDV ORF024 homologue VACV F9L gene, has been characterized as a membrane protein associated with the IMV in VACV, playing a role in cellular entry and fusion as one of the 11 proteins of the entry–fusion complex (EFC) [72,73]. Rabbit polyclonal antibodies generated against F9 demonstrated neutralizing activity against the IMV [72]. Although studies demonstrating F9 as a protective vaccine candidate by itself are limited, it has been utilized in multivalent DNA vaccine studies. F9 was administered along with plasmids encoding VACV proteins A4, A27, A33, A56, B5 H3, and L1, and this was able to confer immunity against lethal monkeypox virus challenge in non-human primates [74]. In LSDV, neither antigenicity nor the ability of ORF024 to induce a humoral immune response has been characterized immunologically as of this publication.

### 5.2. LSDV ORF059 (VACV G9R)

G9R is a VACV gene encoding the 38.8 kDa myristylated protein, G9 [75]. G9 is part of the EFC of VACV that is exposed on the surface and has been shown to be indispensable [76]. In its role as part of the EFC, it may stabilize the A26 protein—a role also attributed to the A16 protein [63,64]. The cowpox homologue of G9 (CPV100) has been found to be a protective immunogen in a mouse challenge model with cowpox and VACV [77]. While there has been some research conducted on the antigenicity of its homologue encoded by LSDV ORF059, as of this publication, the immunogenicity of its homologues along with its exposure on the surface of the IMV leaves room for exploration, as G9R is not fully validated as a protective antigen.

### 5.3. LSDV ORF060 (VACV L1R)

The open reading frame VACV L1R was found to encode a 25 kDa myristylated protein [78]. Early work by Ichihashi and Oie (1996) identified a monoclonal antibody (mAb) (2D5) with the ability to inhibit a “penetration protein” involved in cellular fusion [79]. Other neutralizing antibodies against this protein were also used to map the protein product of L1R to the IMV; furthermore, it was shown to be efficiently extracted using NP40, thus suggesting that it was associated with the surface membranes of IMVs [80]. L1R has been used in the form of a DNA vaccine, as well as an adenovirus-vectored vaccine, where it was able to protect mice from lethal challenge even though the protection was considered partial as the mice exhibited some weight loss following VACV challenge [81,82]. However, when the L1 vaccine was co-administered with A33, all mice exhibited significantly greater protection [82]. This was shown further in a study where non-human primates vaccinated with a combination DNA vaccine containing A27L, A33R, L1R, and B5R genes showed cross-reacting antibodies against all four monkeypox virus orthologues tested [83]. Kaufman et al. [81] also showed the enhanced protective effect of combination vaccines incorporating IMV and EEV proteins. ORF060 from SPPV [70] and LSDV [84] have been expressed in *E*. *coli* and evaluated for immunogenicity in rabbits. The polyclonal antibodies exhibited some level of neutralizing activity at 1:10 for SPPV and 1:28 for LSDV, indicating that this is a potential antigen target for capripoxviruses.

### 5.4. LSDV ORF074 (VACV H3L)

The H3L gene has been characterized as expressing a 35 kDa protein (p35), which was subsequently found to be an antigenic component of VACV [85,86]. It was identified as a detergent-extractable (via NP-40) component of the intracellular mature virion [87]. Further mutational studies found that VACV defective in H3L expression exhibited decreased plaque formation, displaying attenuation when inoculated in mice [88].

Protein microarray experiments covering nearly the entire VACV proteome found that p35 was strongly recognized by human sera following VACV (Dryvax) vaccination [89,90]. Another study using microarrays with a measure of anti-VACV IgG correlating to neutralizing antibody titers in 50 human donors vaccinated with smallpox vaccine found H3 to be ‘the most immunodominant’ target in humans [91]. Moreover, purified anti-p35 antibodies exhibited neutralizing activity, which was present in antibodies from both humans and mice [89]. Polyclonal antibodies generated against the protein also exhibited passive transfer immunity. H3L has also been expressed in *E. coli*, where the purified protein product was then used to vaccinate BALB/c mice (with adjuvant), followed by intranasal challenge with virulent VACV. The H3L-vaccinated mice exhibited significantly greater protection compared with the corresponding negative control [89]. The neutralization of VACV with anti-p35 antibodies has been narrowed down to just seven amino acids, without which neutralizing antibodies were not detected [92]. In LSDV, ORF074 is the homologue of VACV H3L; as of this publication, no immunological characterization has been performed. However, there have been several groups that have developed ELISAs to test the antibody response of small ruminants and cattle against p32 (i.e., the protein product of LSDV ORF074), as it is believed to be immunogenic [67,93,94,95,96]. Most recently, among mAbs produced against an inactivated LSDV Neethling strain, mAbs targeting p32 were preliminarily characterized and selected to develop a competitive ELISA to detect anti-p32 antibodies [97].

### 5.5. LSDV ORF104 (VACV A13L)

VACV A13L is a gene that encodes a 7.7 kDa membrane protein (A13) that is expressed late after infection [98,99]. The C-terminus of A13 was found to be exposed on the surface of VACV [100]. While it has been shown that there was a serological response to A13 in serum samples from vaccinated individuals [91], there have been varying reports on the neutralizing capabilities of antibodies against A13 [100,101]. Unger and Traktman [100] observed anti-A13 rabbit polyclonal serum did not result in any reduction in viral titer. Benhnia et al. [91] reported that there was a low level of IgG response to A13 in Dryvax-immunized donor sera. On the contrary, Xu et al. [101] characterized an anti-A13 mAb that was able to neutralize the VACV IMV. Additionally, the anti-A13 mAb was protective against intranasal VACV challenge in mice and the epitope bound by the neutralizing mAb was determined to be 10 amino acids in length [101]. The conflicting reports on the immunogenicity of A13 add to the need for more conclusive studies on the antigen in capripoxviruses. The neutralizing epitope identified by Xu et al. [101] on the A13 sequence has a 36% sequence identity to LSDV ORF104. A13 has also been identified as 1 of the 15 of 224 VACV antigens that elicited a significant protein microarray antibody response in humans following immunization with modified vaccinia Ankara [90].

While there is little research into LSDV ORF104, there is enough research on the VACV homologue A13L to suggest the potential of the protein to induce neutralizing antibodies and therefore be a vaccine candidate.

### 5.6. LSDV ORF105 (VACV A14L)

VACV A14L encodes a 15 kDa protein (A14) found in viral membranes that has been shown to be important in viral crescent assembly in VACV, as well as in the stable attachment of the viral crescents to viral factory surfaces [102]. The role of A14 in morphogenesis is dependent on phosphorylation by the VACV F10 kinase [103]. This major envelope protein has been shown to be a dominant target of antibodies. While there were no neutralizing antibodies found from mAbs targeting A14, some neutralizing activity was seen in the presence of complement in addition to protection after mice were treated with these mAbs and challenged with the smallpox vaccine [104]. Furthermore, microarray correlation experiments by Benhnia et al. [91] using sera from smallpox vaccine-immunized human donors showed a positive correlation of anti-A14 IgG with neutralizing antibody titers. There is no research regarding the immunogenicity of LSDV ORF105 or ability to elicit neutralizing antibodies as of this publication; however, the work carried out to show the immunogenicity of the VACV homologue is promising.

### 5.7. LSDV ORF109 (VACV A17L)

VACV A17L encodes a 21 kDa protein, A17 (also known as p21), which was initially purified as a co-precipitation product of the 14 kDa VACV protein (p14) encoded by the A27L gene [105]. Early characterization of this protein found that the p14-p21 complex was released upon treatment of VACV with NP-40 detergent. This finding, along with the fact that the sequence of p14 lacked any sizable hydrophobic domains, suggested that p21 served as an anchor for p14 at the surface of the virion [106]. This hypothesis was later confirmed by electron microscopy [107]. Rabbit polyclonal antibodies generated against the N-terminus of p21 were found to neutralize in vitro VACV infection of BSC-40 cells [107]. While antibodies against p21 were generated in humans following vaccination with VACV, no neutralizing antibodies were detected [91]. Nevertheless, the ability to generate neutralizing antibodies against the N-terminus of the protein may suggest that p21 could be used as a vaccine target. In LSDV, A17L is homologous with ORF109 and, as of the submission of this manuscript, no experimental work has been performed to immunologically characterize this protein.

### 5.8. LSDV ORF117 (VACV A27L)

VACV A27L was characterized as an ORF that encodes a 14 kDa envelope protein (p14) [108]. Its localization to the virion envelope was demonstrated using mAbs generated against the protein [108]. Subsequent studies also showed that recombinant p14 could multimerize [109] as well as interact with another VACV protein encoded by the A17L ORF [110]. This, in turn, may also play a role in cellular fusion. It was also shown that A27 is a virulence factor, as VACV variants expressing mutations in A27L showed reduced plaque sizes when propagated in cell culture [108]. The protein product of A27L has demonstrated antigenicity and been a subject of studies showing the induction of protective and neutralizing antibodies developed from the Dryvax smallpox vaccine [91,111,112]. As antibodies generated against the p14 protein were shown to neutralize VACV, its capripoxvirus homologue has been evaluated for immunogenicity. SPPV ORF117 [70] and LSDV ORF117 [84] were expressed in *E. coli*, with polyclonal antisera being generated against the recombinant protein in rabbits. Neutralizing activity was observed at 1:10 for SSPV and 1:8 for LSDV, suggesting that SPPV ORF117 could serve as a potential vaccine target [70,84].

### 5.9. LSDV ORF118 (VACV A28L)

In VACV, A28 (encoded by A28L) was characterized as a membrane-bound component of the intracellular mature virion (IMV), with the 16.3 kDa protein product expressed late during the virus replication cycle [113]. Suppression of A28L expression (through the generation of recombinant VACV, where A28L was inducible via a lac operon) resulted in a conditional lethal phenotype, where the recombinant VACV was incapable of infecting cells in the absence of its expression. Both electron microscopy and treatment of the IMV with detergent demonstrated that the protein product of A28L was membrane-bound [113]. The protein product of VACV A28L was expressed in baculovirus, in a soluble recombinant form, followed by its inoculation in rabbits in order to generate polyclonal antibodies [59]. Upon further analysis, the polyclonal antibody was able to recognize the intact virion, and was also able to neutralize and prevent infection of cells in vitro. Moreover, these neutralizing polyclonal antibodies were able to be used in passive immunization experiments in mice; protection against intranasal challenge with virulent VACV WR was observed in mice previously immunized with purified “A28 serum” [59]. LSDV ORF118 is homologous to VACV A28L and, as of this publication, no immunological characterization has been carried out.

### 5.10. LSDV ORF122 (VACV A33R)

VACV A33R was characterized as an ORF that encodes for a glycosylated protein, A33, that migrated at 23–28 and 55 kDa under reducing and non-reducing conditions, respectively [114]. Four mAbs generated against VACV reacted with this protein, which was also detected following Triton X-114 treatment, thus suggesting that the translated product was an integral membrane protein. Of the four mAbs specific to this glycoprotein, one was also reactive with ectromelia virus (i.e., mousepox), which was found to share 94% amino acid identity with its VACV homologue. Electron microscopy also confirmed that the protein product of A33R localized to the surface of the EEV, but not the IMV [114,115]. Based on this characterization, the ability of A33R to encode for a protective epitope was assayed in multiple antibody and vaccine assays. In vivo mouse studies by Galmiche et al. [116] showed that, while recombinant A33 could protect mice from lethal VACV challenge, the antibodies generated following vaccination were non-neutralizing. This protection is most likely provided through cell-mediated response, which has been observed in capripoxvirus immunizations with live attenuated vaccines, resulting in protection without the induction of neutralizing antibodies [23]. Additionally, the protective ability of A33R was further shown in DNA vaccination studies [82,83,116,117].

LSDV ORF122 is homologous to VACV A33R. The protective properties of ORF122 were assayed in SPPV, where polyclonal antibodies raised against the recombinant protein were assayed for neutralizing activity in vitro. Contrary to its VACV homologue, ORF122 was able to neutralize SPPV infection in lamb kidney cell culture [70]. This also illustrates the importance of performing neutralization studies using capripoxvirus antigens while using information on homologues as a guide to fully understand the immunogenicity of candidate antigens. The neutralization observed in SPPV infection would most likely translate into cross-neutralization in GTPV and LSDV due to the high sequence similarity observed within the capripoxvirus genus.

### 5.11. LSDV ORF123 (VACV A34R)

VACV A34R encodes a 24–28 kDa glycoprotein that was found to be localized to the outer membrane of the EEV [118,119]. Functional studies also demonstrated that A34R was necessary for the detection of VACV-induced actin tails, which are associated with the motility of intracellular enveloped viruses (IEVs) [120]. While there are yet to be neutralizing antibodies characterized for the glycoprotein product of A34R, a homologue of LSDV ORF123, it has been characterized as an immunogenic antigen in capripoxviruses. A truncated protein of GTPV/LSDV ORF123 was found to be a highly sensitive antigen for indirect ELISA detection of LSDV and GTPV/SPPV sera [69]. However, the ability of this protein to induce neutralizing antibodies is not yet clear.

### 5.12. LSDV ORF126 (VACV A36R)

The VACV A36 protein, encoded by ORF A36R, is a 45 kDa partially conserved membrane protein [121,122]. Deletion of A36R resulted in a 5-fold decrease in the EEV and a small-plaque phenotype in VACV, while IMV formation was unaffected [123]. A36 interacts with A33, playing a role in the binding of cellular proteins to facilitate actin tail formation and microtubule movement [124]. However, while the importance of A34 in EEV infectivity has been shown, mAbs against the protein did not show neutralizing activity in an early study [125]. In capripoxviruses, LSDV ORF126 (a homologue of VACV A36R) is believed to be an important antigen, as its modification results in attenuation of LSDV [126] and GTPV [127]. Through in silico analysis, the protein encoded by LSDV126 was found to have a potentially significant antigenic epitope [126]. These findings encourage the exploration of LSDV ORF126 as a vaccine candidate.

### 5.13. LSDV ORF141 (VACV B5R/C3L)

VACV B5R encodes a 42 kDa protein (gp42), which is one of the six proteins found in the EEV envelope [128,129]. Upon its early characterization, the 317-amino acid sequence was found to be homologous to complement control proteins (in particular, C4b-binding protein), as well as VACV C28K, a VACV protein previously characterized as a complement inhibitor [130], suggesting that it may play a role in immune evasion [131]. B5R has also been found to encode a neutralizing epitope, as characterized by Galmiche et al. [116]. In this study, the exposed domain of gp42 was expressed in baculovirus and was used to immunize rabbits. The resulting polyclonal sera were then found to inhibit in vitro plaque formation of VACV [116]. It should also be noted that the recombinant extracellular gp42 was able to be used as a subunit vaccine, protecting BALB/c mice from lethal intranasal VACV challenge. Furthermore, it was also found that the polyclonal antibodies could confer passive immunity in mice against lethal VACV challenge [116].

The VACV C3L gene produces a protein named the vaccinia complement control protein (VCP), which has been characterized as a complement-binding protein [132]. This secreted 35 kDa protein has been shown to affect virulence through modulation of adaptive immunity during infection [133,134]. VCP inhibits host response to infection through inhibitory interactions with complement proteins (C3b/C4b), preventing VACV neutralization [135]. While VCP is a secreted protein and would not allow for neutralization of the virus, it does provide insight to the possible role of the homologous protein in capripoxviruses.

LSDV ORF141 is homologous to both VACV C3L and B5R. This is also the case with M144R (Myxoma virus) and SPV139 (Swinepox virus), which also share homology with both VACV genes [136,137]. Additionally, M144R has been shown to be an essential gene and could play an immunomodulatory role similar to the proteins encoded by VACV B5R and C3L [138]. While there has been no research conducted to date regarding the immunogenicity of LSDV ORF141, the immunogenicity of the homologous proteins makes it a good vaccine candidate and potential target for neutralization.

## 6. Conclusions

### Is One Antigen Sufficient?

Even though single antigens have been shown to elicit neutralizing antibodies against capripoxviruses, the level of protection against an experimental capripoxvirus infection in sheep, goats or cattle is not known. Due to the complexity of poxviruses, a single-antigen vaccine may not provide sufficient protection and it will likely be necessary to incorporate several antigens in a vaccine to elicit full protection against a lethal challenge. With orthopoxviruses, optimal protection required a mixture of mAbs that targeted several membrane proteins, including proteins on the EEV and IMV forms of the virus [47] Furthermore, a combination of four monkeypox virus antigens (A29L, A35R, M1R, and B6R) has recently been shown, in an mRNA vaccine, to protect mice against VACV [139]. Similarly, it is likely that for capripoxviruses, a combination of antigens will provide improved immunity over individual antigens. This will be determined by evaluation of capripoxvirus vaccines containing single and multiple antigens. The selection criteria for determining the combination of capripoxvirus antigens is not straightforward, and a systematic approach of evaluating different numbers and combinations of antigens would be ideal. Furthermore, different antigens could be structured within a single expression system and evaluated for immunogenicity for both antibody responses, including virus neutralization activity as well as T cell responses. In addition, sera specific for individual antigens can be evaluated for neutralization activity and compared to the neutralization activity obtained by combining sera specific to different antigens. While the data available from orthopoxvirus studies provide significant insight to the antigen homologues that have a high potential for immunogenicity and/or neutralizing activity in capripoxviruses, there is a need for specific research to characterize this in capripoxvirus antigens. The results from these experiments will assist in determining the role of multiple antigens in neutralization for capripoxviruses. This review presents a comprehensive analysis of potential protective capripoxvirus antigens based on information from available research and the use of bioinformatics resources. Overall, it is likely that the antigens identified in this paper can be used to develop the next generation of capripoxvirus vaccines. Ultimately, the antigen(s) in different vaccine types will have to be evaluated in the target species using an experimental capripoxvirus challenge to determine the protective antigens.

## Figures and Tables

**Table 1:** Membrane-associated proteins of capripoxviruses. Antigenicity score was obtained using VaxiJen v2.0 software and database. The database identifies proteins with scores of 0.4 and above to represent a probable protective antigen based on the amino acid properties and sequence alignment data with known antigens [42]. Conservation of antigens is presented as full (conservation in all poxviruses) or partial (conservation in chordopoxviruses) based on published characterization [38]. Protein sequence alignments were performed on LSDV Kenya isolate (MN072619.1) with Vaccinia Virus Western Reserve strain (VACV WR), Myxoma virus (MYXV) Lausanne, and Swinepox Virus (SPV) Nebraska using Geneious™ (https://www.geneious.com, 19 February 2025).

LSDV ORF	Protein ID	Antigenicity Score	VACV ORF	Conserved		Essential		Percent Identity (%)		
				Full	Partial		Length (aa)	VACV	MYX	SPV
024	QEJ78569.1	0.4522	F9L	✓		✓	216	44.91	50	64.82
028	QEJ78573.1	0.4289	F13L		✓		370	56.56	73.24	75.41
028.5	QEJ78574.1	0.5536	F14.5L				49	31.25		
038	QEJ78584.1	0.6005	E8R		✓		265	68.42	79.25	80.45
044	QEJ78590.1	0.1969	I2L		✓	✓	72	43.84	52.06	52.06
046	QEJ78592.1	0.3494	I5L		✓		78	29.87	48.72	56.41
052	QEJ78597.1	0.4126	G3L		✓	✓	110	48.65	55.46	55.46
057	QEJ78603.1	0.3041	G7L		✓		373	53.56	58.40	64.66
059	QEJ78605.1	0.3425	G9R	✓		✓	336	45.35	52.38	57.98
060	QEJ78606.1	0.5192	L1R	✓		✓	245	66.12	76.86	86.12
061	QEJ78607.1	0.258	L2R		✓	✓	92	33.33	8.33	92.39
064	QEJ78610.1	0.4574	L5R	✓		✓	131	50.39	54.69	63.28
070	QEJ78616.1	0.6404	J5L	✓		✓	133	58.96	66.92	66.92
073	QEJ78619.1	0.5621	H2R	✓		✓	190	65.61	69.95	73.68
074	QEJ78620.1	0.4302	H3L	✓			322	35.89	54.35	61.92
078	QEJ78624.1	0.5513	H7R		✓	✓	147	42.18	60.54	67.57
100	QEJ78646.1	0.3433	A9L	✓		✓	78	65.39	73.68	84.62
102	QEJ78648.1	0.4551	A11R	✓		✓	317	52.01	73.82	75.24
104	QEJ78650.1	0.4283	A13L		✓	✓	67	31.88	55.88	55.07
105	QEJ78651.1	0.304	A14L		✓	✓	95	55.44	64.95	81.25
106	QEJ78652.1	0.8381	A14.5L		✓		53	58.49	79.25	73.59
108	QEJ78654.1	0.4531	A16L	✓		✓	377	51.70	60.42	66.23
109	QEJ78655.1	0.5584	A17L		✓	✓	196	40	54.90	63.64
113	QEJ78658.1	0.5003	A21L	✓		✓	115	52.24	57.39	62.61
117	QEJ78663.1	0.3768	A27L			✓	148	27.27	35.81	45.95
118	QEJ78664.1	0.4878	A28L	✓		✓	140	51.70	64.29	70
120	QEJ78666.1	0.6266	A30L		✓	✓	74	40.54	57.14	58.67
122	QEJ78669.1	0.4264	A33R				196	27.51	35.57	38.14
123	QEJ78670.1	0.5962	A34R		✓		171	40.70	55.81	57.90
126	QEJ78673.1	0.382	A36R				181	21.16	26.83	26.32
141	QEJ78688.1	0.467	C3L/B5R			✓	225	30.61/23.56	26.50	34.21

**Table 2:** Candidate proteins for next-generation capripoxvirus vaccines. Characterization in LSDV obtained from whole-genome sequence annotation of LSDV Kenya isolate [43]. Transmembrane regions predicted using CCTOP [44].

LSDV ORF	Characterization in LSDV	Function in VACV	Putative LSDV Protein Topology
024	S-S bond formation pathway protein	Component of the entry–fusion complex; incorporated into MV membrane	1TM C terminal; 1–180AA exposed
059	Myristylprotein	Component of the entry–fusion complex; required for entry and membrane fusion	1 TM C terminal; 1–317AA exposed
060	Myristylated IMV envelope protein	Component of the entry–fusion complex; cell entry and membrane fusion	1 TM C terminal;1–181AA exposed
074	IMV envelope protein p32	Antigenic, heparin-binding surface protein	1 TM C terminal; 1–280AA exposed
104	IMV membrane protein	Essential for virion maturation, antigenic	1 TM N terminal; 23–67AA exposed *
105	Phosphorylated IMV membrane protein	Essential for assembly and attachment of viral crescents to virosomes	2 TM; 1–12AA, 66–95AA exposed *
109	Phosphorylated IMV membrane protein	Essential component of nascent viral membranes required to initiate morphogenesis	4 TM; 1–64AA, 106–115AA, 158–204AA exposed *
117	IMV surface protein	Roles in IMV–cell attachment, fusion, microtubule transport	No TM
118	Entry–fusion complex component	Required for cell entry and fusion; essential component of the virion membrane	1 TM N terminal; 22–140AA exposed
122	EEV phosphoglycoprotein	Involved in CEV–cell adherence and actin tail formation	1 TM N terminal; 68–196AA exposed
123	EEV protein	Required for infectivity of EEV	1 TM N terminal; 39–171AA exposed
126	Membrane protein	Integral membrane protein; present on IEV but not IMV or CEV	1 TM N terminal; 25–181AA exposed *
141	EEV host range protein	Contains complement control module	1 TM C terminal; 1–189AA exposed

* Topology results without overwhelming consensus of predicted structure.

**Table 3:** LSDV ORFs encoding membrane-associated proteins that are not well studied in terms of immunogenicity but could be further researched to elucidate antigenicity. Characterization in LSDV obtained from whole-genome sequence annotation of LSDV Kenya isolate [43].

LSDV ORF	Characterization	Function in VACV	Reference(s)
028.5	IMV protein	Involved in virulence and cell adhesion	[45,46]
043	Core protein	DNA binding; shown to induce neutralizing antibodies	[47,48,49]
046	IMV membrane protein	Shown to increase virulence and virus proliferation	[50]
052	Entry–fusion complex component	Essential for entry into host; important in assembly of EFC and stability	[51,52]
057	Virion structural protein	Associated with IMV and cores of mature virion; associates with A30; plays a role in morphogenesis	[53,54]
061	Crescent formation protein	Involved in crescent membrane formation and elongation; may enable stable association of membrane proteins to viral membrane	[55,56]
064	Membrane protein	Expressed late after infection; the C terminus is exposed on the surface	[57]
073	Entry–fusion complex component	Required for virus entry into cells; lethal mutation resulted in attenuation; similar to A28 in structure	[58,59]
100	IMV membrane protein	Expressed late in infection	[60]
102	Viral membrane formation protein	Coimmunoprecipitated with A32; not associated with membranes but easily detected in extracts of VACV-infected cells	[61]
108	Entry–fusion complex component	Found in IMV with N terminus exposed on surface; interacts with G9, A26, and A56.	[62,63,64]
113	Entry–fusion complex component	Found on the lipoprotein membrane of IMV; potentially immunogenic	[47,65,66]

## Data Availability

The original contributions presented in this study are included in the article. Further inquiries can be directed to the corresponding author.
